# Digital health communication and health literacy in times of COVID-19. Planning and implementation of a special course of study in health promotion and prevention

**DOI:** 10.3205/zma001427

**Published:** 2021-01-28

**Authors:** Kevin Dadaczynski, Daniel Tolks

**Affiliations:** 1 Hochschule Fulda, Fachbereich Pflege und Gesundheit, Fulda, Germany; 2 Leuphana Universität Lüneburg, Zentrum für Angewandte Gesundheitswissenschaften (ZAG), Lüneburg, Germany; 3 Klinikum der Universität München, LMU München, Insitut für Didaktik und Ausbildungsforschung in der Medizin, Munich, Germany

**Keywords:** health literacy, health communication, COVID-19, MS teams, online Inverted Classroom, audience response systems

## Abstract

As a result of the corona pandemic, the amount of digital health information has increased substantially. As the quantity and diversity of information increased, so does the need for evidence based and reliable health information. In the special course of study “Health Communication”, students of the Bachelors program “Health Promotion” at Fulda University of Applied Sciences are enabled to develop and disseminate evidence-based health information and preventive messages that meet the demands of the target group. Due to the corona-related university closure, the module “Digital Health Communication” was realized in a digital format during the summer semester 2020. In order to activate students and promote teamwork, the study course used the approach of problem-based and research-based learning. Moreover, the course concept is based on a variety of methods, including MS Teams with screencasts, videos, synchronous teaching sessions, gamified audience response systems, the online Inverted Classroom Model and a final oral examination. Despite various challenges such as the short planning period or the necessary restructuring of a part previously planned as “en bloc”, the experiences are mostly positive. Among other things, the use of MS Teams as an integrated learning, collaboration and communication platform has proven to be useful. In the students' feedback, the broad use of methods, the gamification elements and the flexibility of the lecturers are evaluated positively.

## Zusammenfassung

Infolge der Corona-Pandemie ist insbesondere das digitale Informationsaufkommen zum Thema Gesundheit substantiell angestiegen. Mit zunehmender Informationsvielfalt steigt der Bedarf an abgesicherten Gesundheitsinformationen. Im Studienschwerpunkt „Gesundheitskommunikation“ werden Studierende der Gesundheitsförderung an der Hochschule Fulda in der evidenzbasierten und zielgruppengerechten Gestaltung und Verbreitung von angemessenen gesundheitsbezogenen Informationen und präventiven Botschaften befähigt. Aufgrund der coronabedingten Hochschulschließung wurde das Modul „Digitale Gesundheitskommunikation“ im Sommersemester 2020 ausschließlich digital umgesetzt. Zur Aktivierung und zur Förderung der Teamfähigkeit orientiert sich das Modul am Ansatz des problembasierten und forschenden Lernens. Zudem beruht das Kurskonzept auf einem Methodenmix, basierend auf MS Teams mit Screencasts, Videos, synchronen Sessions, gamifizierten Audience Response Systemen, dem online Inverted-Classroom-Modell und einer abschließenden Präsenzprüfung. Trotz Herausforderungen, z. B. infolge der geringen Planungszeit oder der notwendigen Umstrukturierung einer Blockveranstaltung sind die Erfahrungen überwiegend positiv. Als sinnvoll hat sich u. a. der Einsatz von MS Teams als integrierte Lern-, Kollaborations- und Kommunikationsplattform erwiesen. In den Rückmeldungen der Studierenden werden vor allem der breite Methodeneinsatz, die spielerischen Elemente und die Flexibilität der Lehrenden positiv bewertet.

## Introduction

Since January 2020, more than 429,000 people in Germany have been diagnosed with SARS-CoV-2 (Severe Acute Respiratory Syndrome Coronavirus 2), of which about 10,000 patients have died (as of October 2020, [1]). Germany has made numerous efforts to manage and control the outbreak, following the recommendations of the National Pandemic Plan COVID-19 [2], [3]. In addition to diagnostics, case identification and measures on infection hygiene measures, these include a broad range of activities to communicate health information, e.g. on symptoms of the COVID-19 disease, protective behavior, support services and legal regulations. As a result of the increasing availability and use of digital technologies and media, communication takes place primarily via digital sources and channels. In this context, information is not only disseminated by public institutions or professional experts, but also processed communicatively by a wide range of recipients (e.g. comments on social media). The continuous improvement of knowledge about COVID-19 as well as the possibility to actively participate in digital communication independent of time and place have led to an increase in the amount of information available within a short period of time. This phenomenon of information overload is increasingly referred to as infodemic [4]. Against the background of possible adverse health effects of false and misinformation [5], [6], prospective Public Health professionals must be enabled to develop and disseminate evidence-based and targeted health information and preventive messages during their studies. 

**Figure 1:**
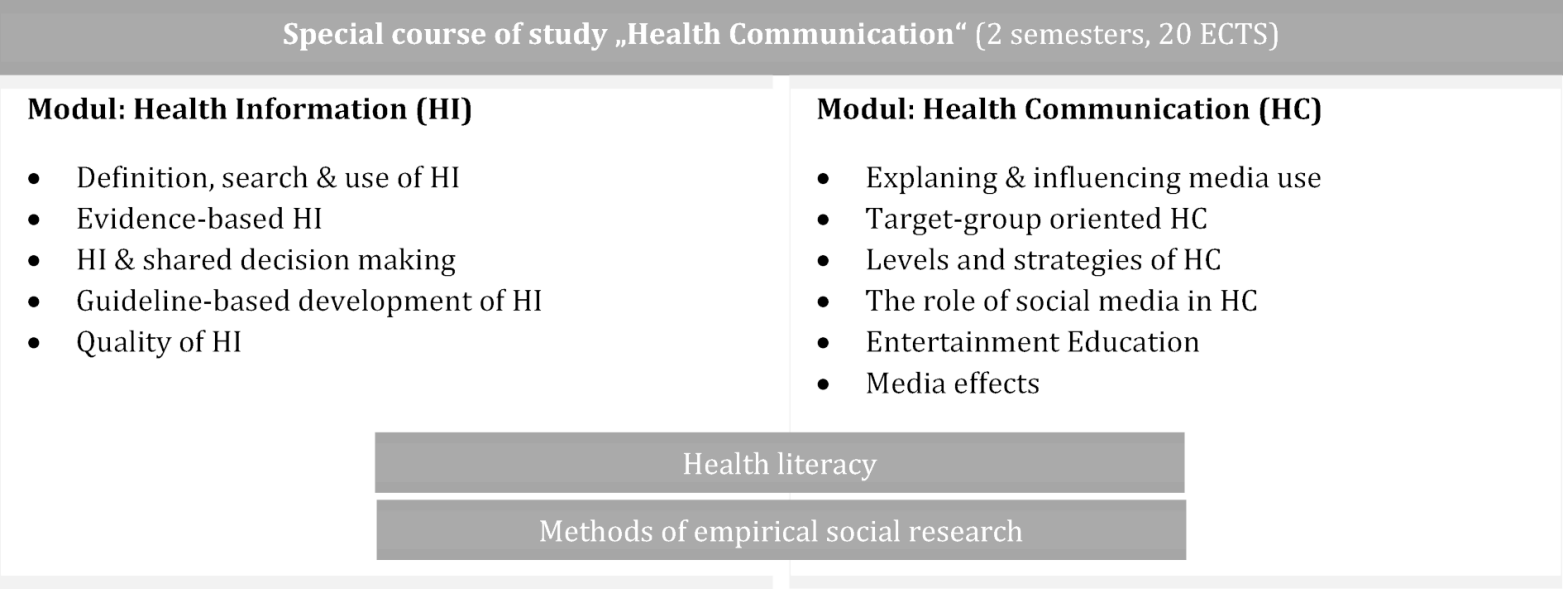
Overview of the special course of study “Health Communication”

At Fulda University of Applied Sciences, health communication is one of the main areas of study in the Bachelor‘s program in Health Promotion. According to Rossmann [7] health communication can be defined as a field of research and application, “[...] which deals with the social conditions, consequences and meanings of health-related and health-relevant, intended and unintended, intrapersonal, interpersonal, media and public communication”. The special course of study lasts over two semesters, with the first semester focusing on evidence-based health information. In the second semester an introduction to digital health communication is given. In addition to learning and applying methods of empirical social research, health literacy is understood as a cross cutting issue. Health literacy refers to people's knowledge, motivation and ability to access, understand, appraise, and apply health information to promote, maintain and restore one’s own health [8] (see figure 1 (Fig. 1)). 

## Planning and digital implementation under corona conditions

The teaching module “Health Communication in Digital Media” is based on the concept of problem-based and research-based learning, starting from a comprehensive topic for which a communication concept has to be developed in small working groups. The concept should (1) address different levels of communication (e.g. mass communication and individual communication) and media (e.g. social media, blog, podcast) (2), take current quality requirements into account and (3) not exceed a defined fictional budget. Next to the overall concept, first insights into the implementation via media snippets (e.g. teaser of a podcast or a social media account) should be given. The students were provided with a model with nine work steps to complete the task. After an initial phase of brainstorming, the steps included a target group analysis and a media analysis as well as the definition and theory-based development of communication levels, formats and strategies. 

Due to the corona-related university closure, the teaching module “Digital Health Communication” was realized in a digital format using the platform Microsoft (MS) Teams. The main reasons for choosing this platform were the different possibilities for collaboration and the use of various communication tools (see table 1 (Tab. 1)). Due to problems with the quality of internet speed, the initial phase of the semester was mainly characterized by asynchronous teaching and learning scenarios (lessons, videos). However, during the course of the semester, synchronous formats could increasingly be integrated via MS Teams and Cisco WebEx (group meetings, discussions of teaching units). The Online Inverted Classroom Model was also applied [9], in which students had to complete various online self-study phases to acquire knowledge of the facts. In subsequent synchronous sessions, the content was applied by the students and deepened in accordance with Bloom's taxonomy [10]. To promote the activation of the students according to the ICAP model [11], we used audience response systems such as Kahoot! [12].

## Experiences

Two of the biggest challenges were the short time span to develop a course concept implemented exclusively in a digital format and the limited possibilities for student activation. In addition, a block seminar originally designed for three days (by an external lecturer) as part of the teaching module had to be spread over two weeks (through screencasts and synchronous teaching), resulting in a higher workload for the students. Working with MS teams proved to be advantageous, as students were able to use different ways of communication (chat, video) as well as self-determined cooperation in the groups (own group channel with project management tools) integrated in one platform. Hence, our experience confirms the results of a recently published scoping review, which emphasizes the importance of self-directed digital learning [13]. However, the use of MS teams was associated with some difficulties. For example, the integration of external lecturers turned out to be complex, some features or applications could not be used or only to a limited extent. Cross-university cooperation with MS teams is therefore currently still difficult. However, a process of reinforcement also became clear, i.e. already active and high-performing students had hardly any adaptation problems, while rather weaker students were even less involved in the course. This required a higher degree of support from the teaching staff (e.g. through regular meetings with the groups, chats and small-step feedback processes).

Despite various challenges posed by the COVID-19 pandemic, the experience in implementing this teaching module has been positive (see table 2 (Tab. 2)). The mix of methods used made it possible to better address the different learning preferences of the students. These efforts are reflected in the positive feedback from the students, which was collected using a mixed-method approach, including quantitative course evaluation, short feedback questions via MS teams and oral discussion groups. In addition to the general digital format, the use of playful elements and the flexible implementation were rated positively. In the forthcoming semesters, the course concept can be used as an online-only teaching module or further developed as a blended learning format.

## Competing interests

The authors declare that they have no competing interests. 

## Figures and Tables

**Table 1:**
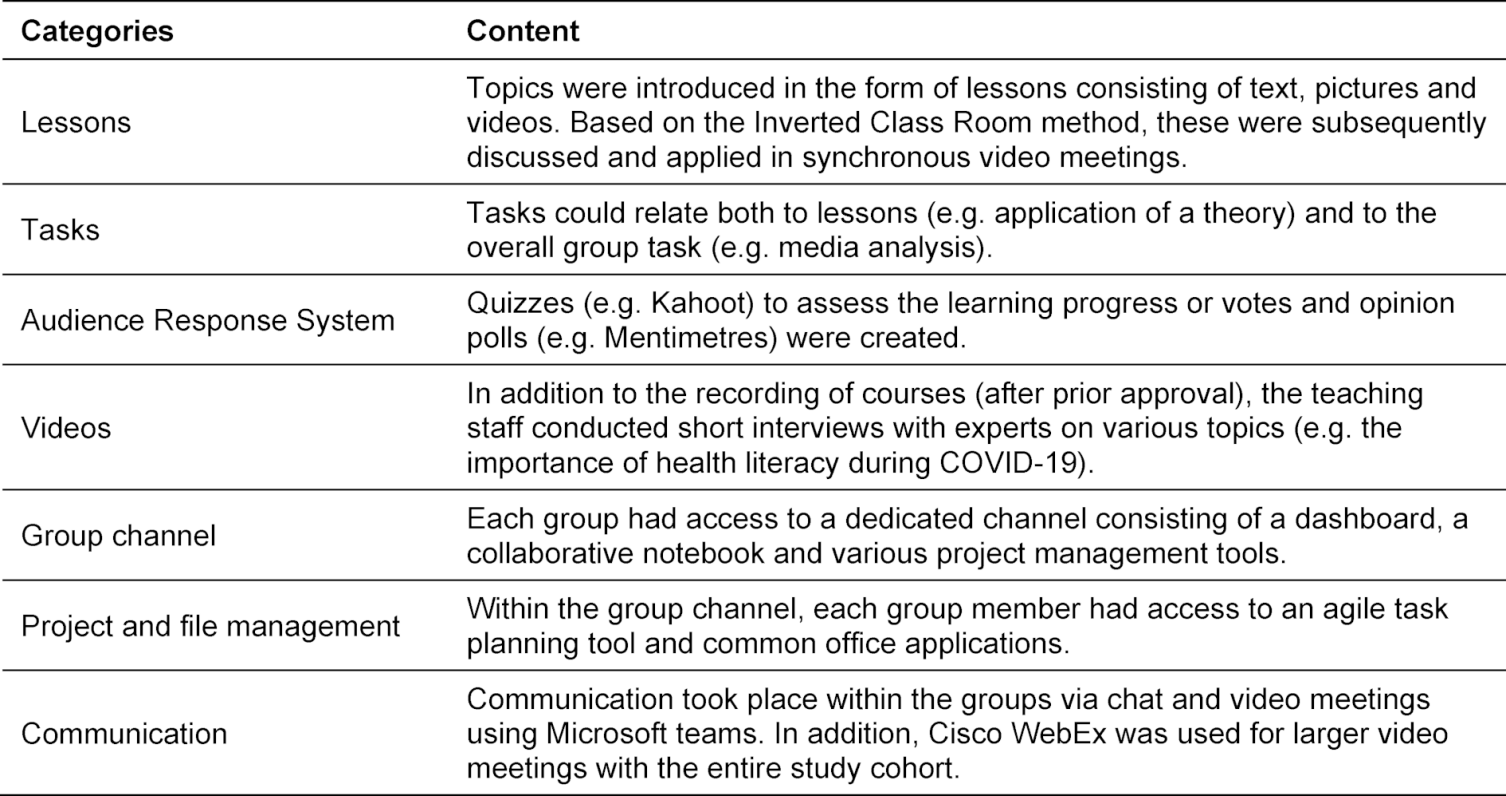
Methods used for the implementation of the teaching module “Health Communication”

**Table 2:**
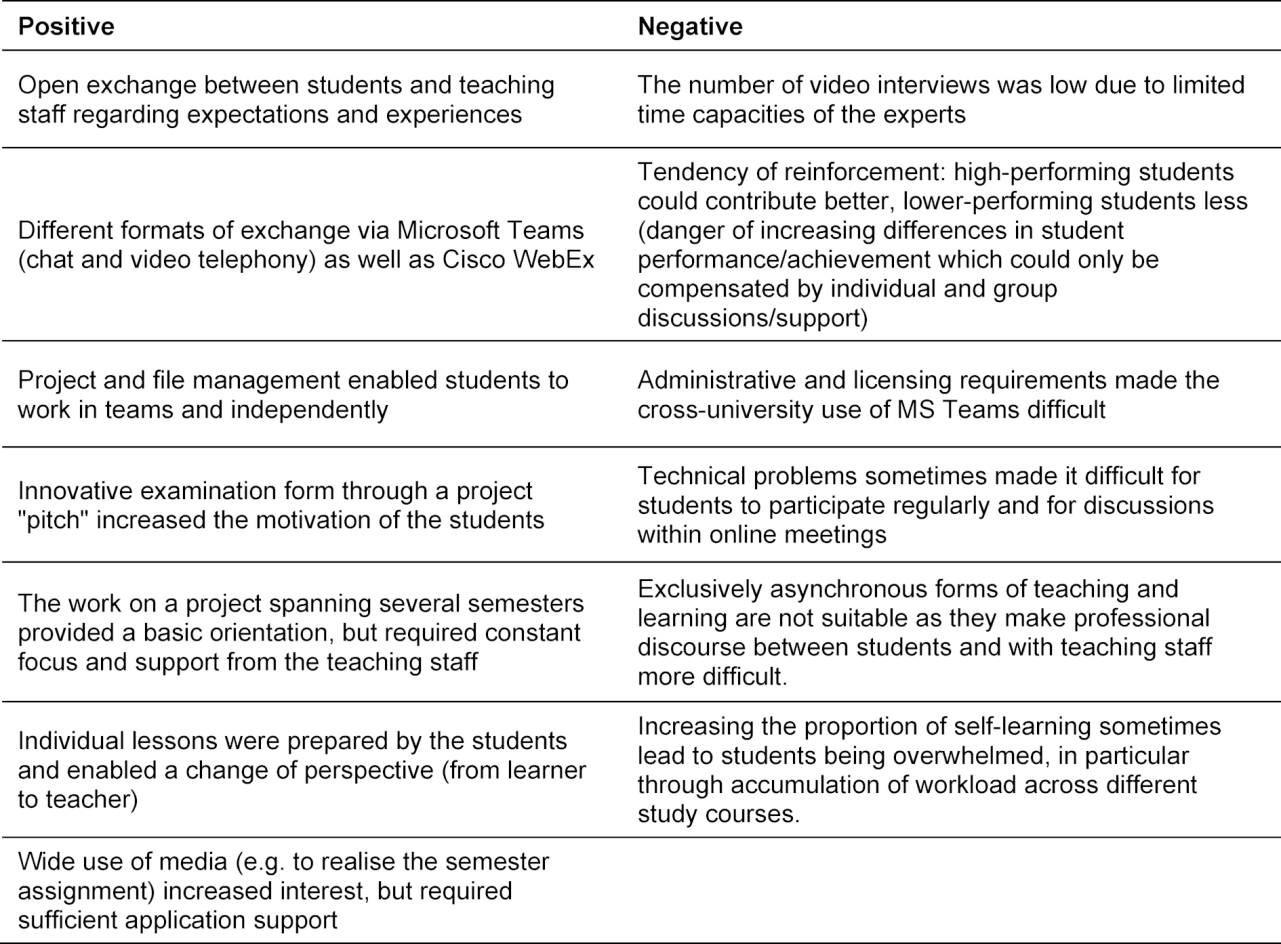
Positive and negative experiences during the teaching module “Health Communication”
